# ATAC-seq and RNA-seq analysis unravel the mechanism of sex differentiation and infertility in sex reversal chicken

**DOI:** 10.1186/s13072-022-00476-1

**Published:** 2023-01-09

**Authors:** Xiuan Zhang, Jianbo Li, Xiqiong Wang, Yuchen Jie, Congjiao Sun, Jiangxia Zheng, Junying Li, Ning Yang, Sirui Chen

**Affiliations:** National Engineering Laboratory for Animal Breeding and Key Laboratory of Animal Genetics, Breeding and Reproduction, Ministry of Agriculture and Rural Affairs, China Agricultural University, Beijing, China

**Keywords:** Chicken, Sex determination and differentiation, Sex reversal, RNA-seq, ATAC-seq, Infertility

## Abstract

**Background:**

Sex determination and differentiation are complex and delicate processes. In female chickens, the process of sex differentiation is sensitive and prone to be affected by the administration of aromatase inhibitors, which result in chicken sex reversal and infertility. However, the molecular mechanisms underlying sex differentiation and infertility in chicken sex reversal remain unclear. Therefore, we established a sex-reversed chicken flock by injecting an aromatase inhibitor, fadrozole, and constructed relatively high-resolution profiles of the gene expression and chromatin accessibility of embryonic gonads.

**Results:**

We revealed that fadrozole affected the transcriptional activities of several genes, such as *DMRT1*, *SOX9*, *FOXL2*, and *CYP19A1*, related to sex determination and differentiation, and the expression of a set of gonadal development-related genes, such as *FGFR3* and *TOX3*, by regulating nearby open chromatin regions in sex-reversed chicken embryos. After sexual maturity, the sex-reversed chickens were confirmed to be infertile, and the possible causes of this infertility were further investigated. We found that the structure of the gonads and sperm were greatly deformed, and we identified several promising genes related to spermatogenesis and infertility, such as *SPEF2*, *DNAI1*, and *TACR3*, through RNA-seq.

**Conclusions:**

This study provides clear insights into the exploration of potential molecular basis underlying sex differentiation and infertility in sex-reversed chickens and lays a foundation for further research into the sex development of birds.

**Supplementary Information:**

The online version contains supplementary material available at 10.1186/s13072-022-00476-1.

## Background

Chickens are important agricultural animals and have been regarded as major food sources for humans for decades [1–3]. The global poultry industry has recently sought effective methods for sex modulation in chickens for different purposes. Particularly in the egg industry, only female chickens are needed, and males are usually cruelly culled, seriously violating animal welfare precepts and causing economic losses [4–8]. Deciphering a mechanism for sex determination and differentiation in chickens would contribute to the prevention of these issues and the research of sex control technologies in the poultry industry. In addition, the high accessibility of gonads makes chicken embryos perfect models to study vertebrate sex determination and even certain human sex differentiation disorders, which will not only enrich the knowledge base of research on vertebrate sex development but also offer ideas for disease treatment [9–17]. However, the paucity of knowledge of chicken gonadogenesis and sex development limits our ability to perform further studies. Thus, it is imperative to explore the specific mechanisms of chicken gonad development as well as sex determination and differentiation.

In chicken embryos, gonadogenesis occurs on approximately embryonic Day 3 (E3) of development (Hamburger Hamilton Stage 20, HH20) [18]. At this time, undifferentiated and indistinguishable gonads, located on the ventromedial surface of the embryonic mesonephros, initially comprise an outer layer of the coelomic epithelium, the cortex, and underlying cords of somatic cells, the medulla [19, 20]. As development progresses, the morphological difference in bilateral gonads is first visible histologically on E5.5 (HH28) [20]. Inside the gonad, primordial germ cells (PGCs), which have migrated into gonads via the bloodstream from the extraembryonic germinal crescent, are the most important cells [21]. In male embryos, PGCs condense into medullary cords surrounded by Sertoli cells, and the hypo-proliferating cell population of the outer epithelial layer does not contain any PGCs [22]. The PGCs differentiate into spermatogonia in the embryonic testes and enter a resting phase until 10 weeks after hatching [23]. Spermatogenesis occurs when chickens are sexually mature, which leads to the development of mature sperm [23]. However, in female embryos, the cortex layer, especially in the left gonad, progressively thickens and contains a large number of PGCs, but the inner medulla becomes vacuolized and forms characteristic fluid-filled cavities [24–26]. The right gonad is encircled by a thin, flat epithelial layer and enters developmental arrest in later stages [25]. Female PGCs in the cortex start differentiating into primary oocytes in the left ovary on E8 (HH34), and first enter meiosis on E13 (HH39) which stops just after hatching [27]. Interestingly, the process of gonadogenesis in sex-reversed chickens is opposite to that of their genetic sex [28, 29]. For instance, treatment with oestradiol causes male embryo feminization and induces the asymmetric development of gonads similar to that in females, characterized by a thickened gonadal cortex and fragmented medulla [30, 31]. In contrast, injection of the aromatase inhibitor fadrozole, which significantly blocks the production of oestrogen, can lead to the permanent masculinization of genetic females, which is characterized by the differentiation of testis-like structures surrounded by thin cortices and a well-organized medulla in the gonad [32–36]. Moreover, in fadrozole-treated sex-reversed chickens, PGCs develop in the medullary cords, not the cortex, and can produce normal Z-bearing sperm through meiosis and underdeveloped W-bearing spermatozoa [37, 38]. Although the systematic developmental processes of embryonic chicken gonadogenesis and gametogenesis are relatively clear and multiple studies have identified the regulatory programs during chicken sex reversal to a certain extent, the underlying functional drivers and key genes remain to be further explored [35, 39].

Previous studies have confirmed that the downregulated expression of key female sex differentiation marker genes after treatment with fadrozole was accompanied by the upregulation of testis development-related genes [33]. For instance, *DMRT1*, which is highly expressed in the gonadal medulla of male chickens and is involved in testicular development by activating the downstream male sex-related genes *SOX9* and *AMH*, is significantly upregulated during sex reversal [40–42]. In addition, the expression of several genes was downregulated in sex-reversed chicken gonads, such as *FOXL2* and *CYP19A1*, which have been reported to be detected in female gonads in a sexually dimorphic manner [42–44]. Despite the recent identification of putative candidate genes, the mechanisms by which certain genes regulate sex differentiation and gonadogenesis remain unclear. Noticeably, our previous studies have revealed that epigenetic modification contributed to chicken sex differentiation by influencing the expression of sexual dimorphism genes [35, 45]. In recent years, accessible regions bound by transcription factors (TFs) have been considered to be the reason for differences in gene expression and were involved in further determining gonadal fates [46–49]. Therefore, based on the relationship between chromatin accessibility and gene expression, it is important to understand the dynamic patterns and regulatory functions of open chromatin regions in chicken sex differentiation and sex reversal.

In this study, to better understand the mechanisms underlying sex determination and differentiation in chickens, we explored the transcriptional and epigenetic differences between wild-type and sex-reversed chicken gonads by performing RNA sequencing (RNA-seq) and Assay for Transposase Accessible Chromatin sequencing (ATAC-seq). The results revealed an effect of fadrozole on the development of chicken gonads. In the present work, we discovered the relationship between gene expression and chromatin accessibility during chicken sex reversal, and identified that the expression levels of numerous candidate genes and cis-regulatory elements, such as *DMRT1*, *SOX9*, *TOX3*, *FOXL2*, *CYP19A1*, and *FGFR3*, were regulated by epigenetic modification at the time of sex differentiation. Furthermore, we found that fadrozole permanently influenced the differentiation of secondary sexual characteristics and reproductive tissues, and we identified several candidate genes, such as *SPEF2*, *DNAI1*, and *TACR3*, that were related to infertility in sex-reversed chickens. In summary, our findings provide critical insights into the sex development, especially the sex reversal, of birds, and will be beneficial for the research of sex modulation in the poultry industry and the treatment of human sex disorders.

## Results

### Transcriptional profiling of embryonic chicken gonads

**Fig. 1:**
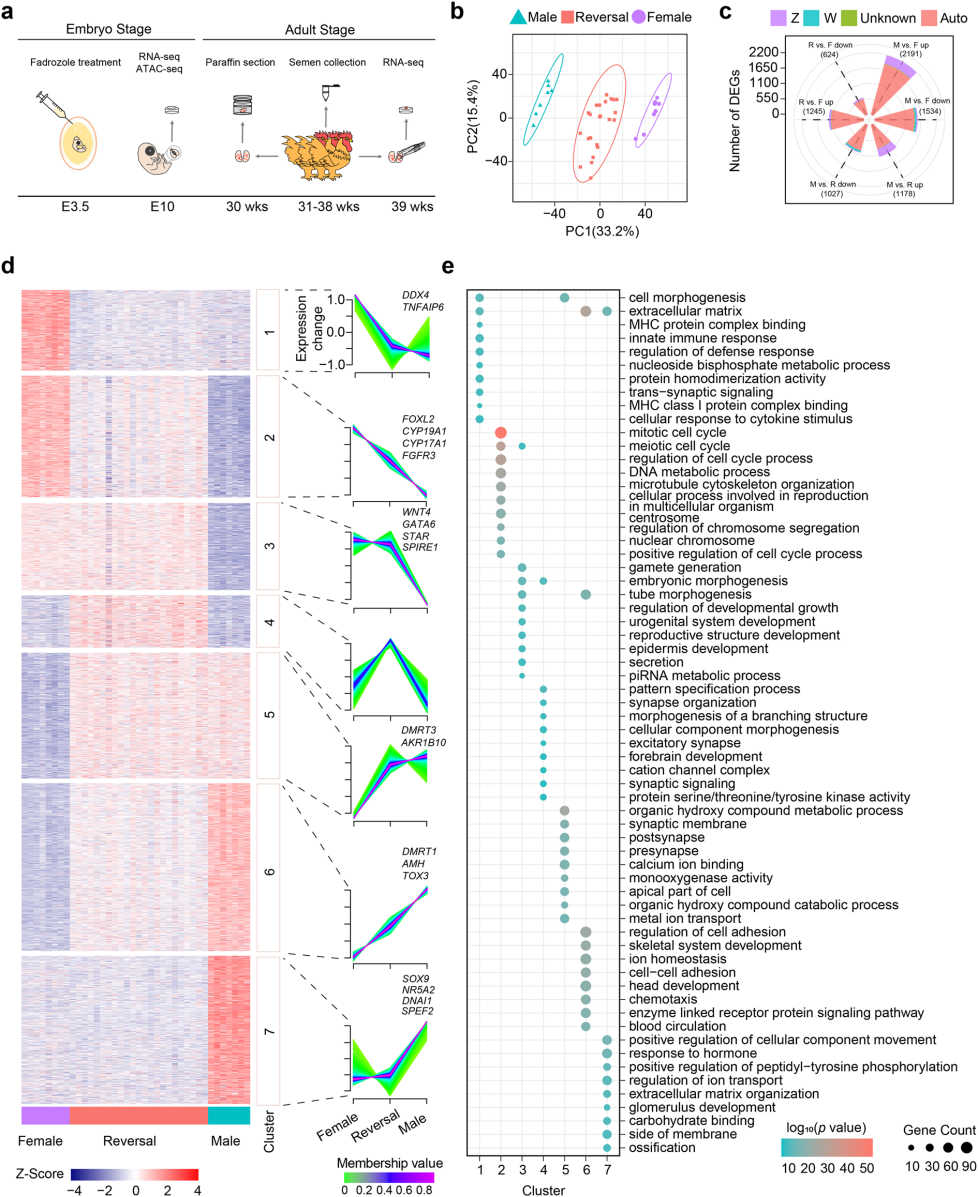
Analysis of RNA-seq data from embryo chicken left gonads**. a** Schematic of experiments done in embryo and adult chicken. The developmental stages are shown below. **b** PCA plot of RNA-seq data from embryo chicken left gonads.** c** Number of DEGs and chromosomal allocation of embryo chicken left gonads, including three pairwise comparisons: male vs. female (M vs. F), male vs. reversal (M vs. R), reversal vs. female (R vs. F). Since male chicken do not have W chromosomes, the expressed genes on W chromosomes are marked as “down-regulation” in M vs. F and M vs. R. **d** Expression profiles of DEGs in three pairwise comparisons. Left: DEGs expression heatmap. Right: seven clusters of DEGs revealed by fuzzy c-means algorithm. The x axis represents each group, while the y axis represents log2-transformed, normalized intensity ratios in each group. **e** Top ten significantly enriched Gene Ontology terms for DEGs in each cluster

To determine systematic differences in the transcriptome landscape between embryonic chicken gonads treated with and without fadrozole, RNA-seq was performed on E10 chicken left gonad samples taken from male chickens (M, *n* = 7), female chickens (F, *n* = 8) and sex-reversed chickens (R, *n* = 23) (Fig. 1a). The number of clean reads and the mapping statistics of each sample were displayed in Additional file 4: Table S1, which showed the high reliability of the data. A Pearson’s correlation heatmap illustrated high correlation within each group (Additional file 1: Fig. S1a). A principal component analysis (PCA) was carried out based on the gene expression landscape, and organized the replicates into three distinct groups corresponding to the M, R and F conditions, with PC1 representing 33.2% of the variance in the data (Fig. 1b). To identify differentially expressed genes (DEGs), we performed pairwise differential expression analysis with the three groups. The DEGs were shown in Fig. 1c and Additional file 1: Fig. S1b, c. The majority of the DEGs were located on autosomes (87%), and nearly 10% of the DEGs were on the Z chromosome. Interestingly, we found that the comparison between M and F led to a larger number of DEGs than that between M and R (Additional file 1: Fig. S1d). This difference was mainly reflected in the DEGs located on autosomes, indicating that fadrozole reduced the gap in the differences between genetic males and females and caused female chicken masculinization.

The DEGs in all the groups were clustered using the fuzzy c-means algorithm. In total, seven different clusters of genes were identified based on their differential expression levels among the M, R and F groups (Fig. 1d; Additional file 4: Table S2). When compared to those in the female group, a total of 1,170 DEGs (454 and 716 in Clusters 1 and 5, respectively) were significantly downregulated or upregulated, and these DEGs were expressed at similar levels between the sex-reversed and male groups. The female development-related genes *DDX4* and *TNFAIP6* and the male development-related genes *DMRT3* and *AKR1B10* were among the DEGs expressed at similar levels (Fig. 1d). A functional enrichment analysis indicated that the DEGs in Clusters 1 and 5 were mainly involved in cell morphogenesis, extracellular matrix, organic hydroxy compound metabolic process, and organic hydroxy compound catabolic process pathways (Fig. 1e). Interestingly, we identified a total of 1,634 DEGs (687 and 947 in Clusters 2 and 6, respectively) that were expressed at an intermediate level in the sex-reversed group (Fig. 1d). In Cluster 2, the expression of the DEGs in the sex-reversed group was significantly lower than that in the female group but higher than that in the male group. These genes included *FOXL2*, *CYP19A1*, *CYP17A1*, and *FGFR3*, which were enriched in the meiotic cell cycle and cellular processes involved in reproduction in multicellular organism pathways and played important roles in female sex differentiation (Fig. 1d, e). However, the DEGs in Cluster 6 presented completely opposite expressional patterns than those in Cluster 2. These DEGs included a set of male sex differentiation-related genes, such as *DMRT1*, *AMH*, and *TOX3,* which were involved in processes, such as tube morphogenesis (Fig. 1d, e). Moreover, in Clusters 3 and 7, the expression of a number of DEGs (491 and 836, respectively) showed no change between the female and sex-reversed groups. This gene set included *WNT4*, *GATA6*, *NR5A2*, *SOX9*, and *DNAI1*. (Fig. 1d). A functional analysis indicated that the DEGs in Clusters 3 and 7 were mainly enriched in gamete generation, reproductive structure development, response to hormone, and glomerulus development pathways (Fig. 1e). These results suggested that fadrozole regulated functional gene expression at different levels, affected the phenotype acquisition, and induced physical changes in the sex-reversed group.

### Analysis of chromatin accessibility in embryonic chicken gonads

**Fig. 2:**
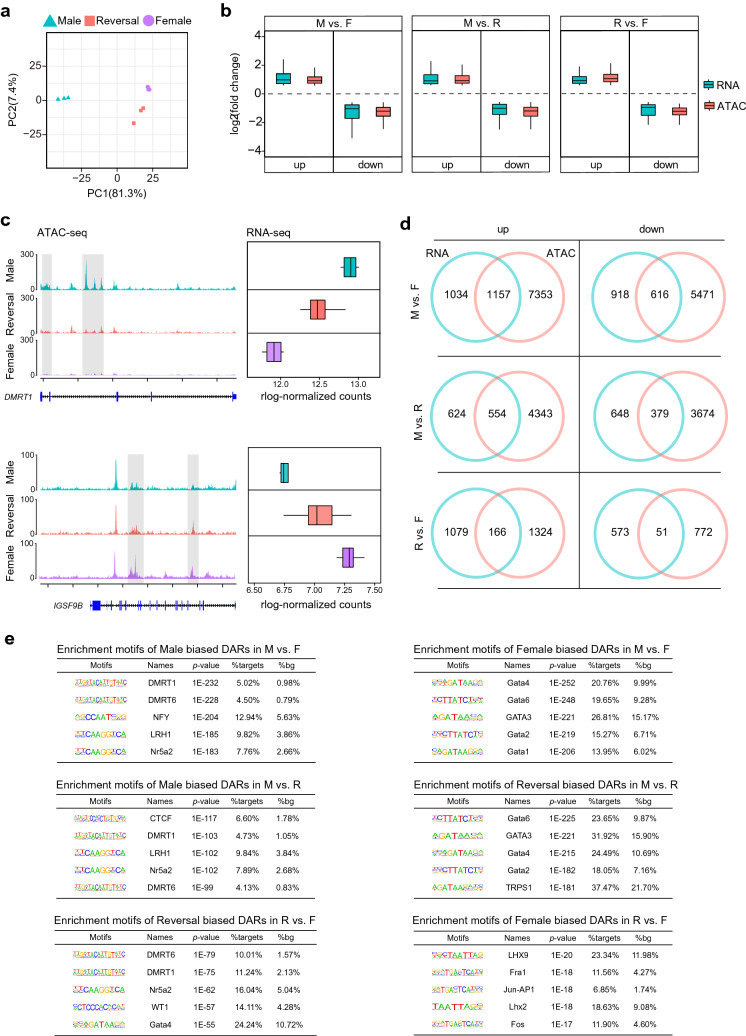
Analysis of ATAC-seq data from embryo chicken left gonads. **a** PCA plot of ATAC-seq data from embryo chicken left gonads. **b** Correlation between DEGs and DARs in three pairwise comparisons. **c** Example of DEGs (DMRT1) showing gradually increasing expression pattern during female to male transition and their associated DARs, as well as DEGs (IGSF9B) showing gradually decreasing expression pattern during female to male transition and their associated DARs. ATAC-seq tracks are shown in the RPKM scale. The y axis of the RNA-seq boxplot shows their group name, and the x axis shows the mean rlog-normalized counts. **d** Venn diagrams show the shared and unique genes obtained from RNA-seq and ATAC-seq in three pairwise comparisons. **e** Top five enriched motifs in different biased peaks in three pairwise comparisons

To explore changes in accessible regions in the genome after treatment with fadrozole, we performed an ATAC-seq analysis with the three groups. The mapping rate of the sequencing data is shown in Additional file 4: Table S3. A Pearson’s correlation heatmap illustrated that replicates were highly consistent within each group (Additional file 2: Fig. S2a). A PCA plot showed that genetic males and females could be divided into two clusters on the basis of PC1 (81.3%), and the three groups could be clearly separated on the basis of PC2 (7.4%) (Fig. 2a).

Subsequently, we performed pairwise comparative analysis based on the ATAC-seq data obtained from the three groups to evaluate the differentially accessible regions (DARs). The results were shown in Additional file 2: Fig. S2b, c. The DARs between the female and sex-reversed chickens were mainly located on autosomes, and nearly 4% of the reversal-biased DARs were located on the Z chromosome. Then, we annotated the DARs onto the genome, which was divided into promoter, exon, intron, and distal intergenic regions, to identify their genomic distribution. We found that gene body regions (promoter, exon, and intron regions) harbored nearly 65% of the DARs in the R vs. F comparison. Approximately 50% of the male-biased DARs were located in promoter regions compared with those of the female or sex-reversed chickens (Additional file 2: Fig. S2c).

To establish the relationship between changes in chromatin accessibility and gene expression, we combined ATAC-seq with RNA-seq. As expected, we found a strong correlation between the DARs and DEGs in all groups by calculating fold changes after assigning open chromatin regions to the nearest DEGs (Fig. 2b). The expression of *DMRT1* was gradually upregulated during the female-to-male transition and was accompanied by increased chromatin accessibility (Fig. 2c). In contrast, *IGSF9B* was highly expressed in the female chickens but was gradually reduced in the sex-reversed and male chickens, and was in parallel with the marked decrease in chromatin accessibility (Fig. 2c). These results suggested a strong link between chromatin accessibility and the expression of genes in embryonic chicken gonads.

To better understand how fadrozole influences gene expression, we integrated DEGs with DARs into different groups (Fig. 2d; Additional file 4: Table S4). As the Venn diagrams show, we found 1,157 upregulated DEGs carrying DARs, including genes related to male sex development, such as *DRMT1*, *SOX9*, *TOX3*, *AMH*, and *NR5A2,* in the M vs. F comparison. In addition, 616 downregulated DEGs carried DARs functioning as female sex-related genes, including *FOXL2*, *CYP19A1*, *FGFR3*, *WNT4*, and *GATA6*. Focusing on the impact of fadrozole on chromatin accessibility, we identified 166 upregulated DEGs containing DARs in the R vs. F comparison. Interestingly, *DMRT1*, *TOX3*, and *SOX9* were also included in this group, which further proved that treatment with fadrozole resulted in dynamic changes in the chromatin accessibility of male sex-related genes in sex-reversed chickens. However, only 51 downregulated DEGs carrying DARs were identified in the R vs. F comparison, and they included *FGFR3*, *WNT10A* and *WNT8A*. Moreover, 554 upregulated DEGs carrying DARs were found between male and sex-reversed chickens, including *SOX9*, *AMH*, *NR5A2* and *DNAI1*. In this comparison, *FOXL2*, *CYP19A1*, *CYP17A1*, *FGFR3*, and *GATA6,* were downregulated DEGs with DARs. These results suggested that fadrozole might indirectly influence genomic chromatin accessibility around or inside sex-related genes to regulate their expression in sex-reversed chickens.

According to previous research, fadrozole could competitively inhibit the activity of aromatase and reduce the production of oestrogen, which could cause changes in chromatin accessibility and the binding of related TFs [50]. Therefore, to further investigate the exact changes in TFs, which might influence gene expression, after treatment with fadrozole, we performed motif analysis for three different pairwise comparisons with HOMER. In the comparison between males and females, we found that the binding sites of TFs related to male gonadal and sex differentiation, such as *DMRT1*, *DMRT6*, and *NR5A2,* were enriched in male-biased DARs (Fig. 2e). As expected, these loci were also enriched in male-biased DARs in the replicates of the M vs. R groups. Interestingly, the *CTCF* motif, a DNA-binding protein that played a key role in the regulation of chromatin interactions and gene expression via cis-regulatory elements, was also significantly enriched in the male-biased DARs in the M vs. R groups [51, 52]. Compared with those in females, reversal-biased DARs showed significant enrichment of motifs associated with *DMRT1*, *DMRT6*, and *NR5A2*, indicating that these TFs likely contributed to the masculinization of sex-reversed chicken gonads (Fig. 2e). However, the binding loci of the *GATA* transcription factor family were significantly enriched in female and reversal-biased DARs compared with male birds, and *LHX9*, *LHX2*, and *Jun-AP1* motifs were enriched only in female-biased DARs compared with the reversal group (Fig. 2e). Taken together, these results suggested that fadrozole significantly affected the genomic chromatin accessibility and binding of TFs related to sex determination and differentiation, which further influenced the expression of sex development-related genes in sex-reversed chickens.

### Growth performance analysis of adult chickens

Based on the relationship between gene expression and phenotype, we wanted to determine whether the injection of fadrozole during the embryonic period can influence the formation of phenotype of adult chickens. Therefore, we established a sex-reversed chicken group and two control chicken groups, wild-type male and female chickens. The phenotypic sex of the hatchlings was identified by anal swelling, and the genetic sex was determined by PCR. Interestingly, we found that the phenotypic sex of all hatchlings in the sex-reversed group was male. Therefore, the rate of sex reversal was 100% (Additional file 4: Table S5).

**Fig. 3:**
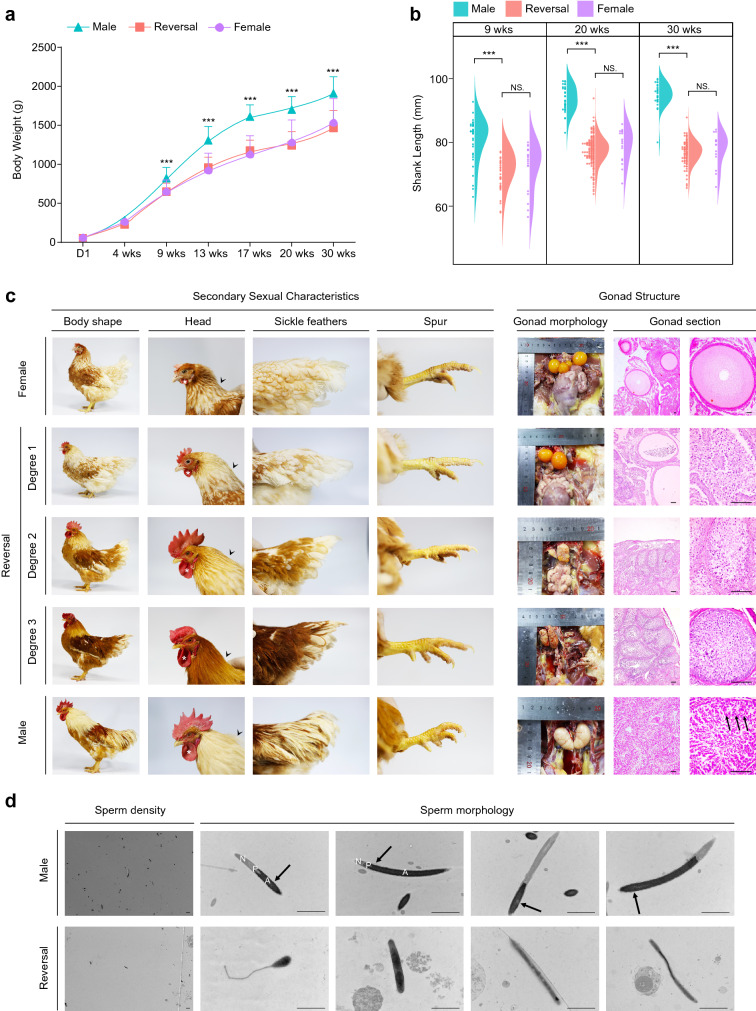
Growth performance and phenotype of adult chicken. **a** Body weight of adult chicken. Asterisks indicate a statistically significant difference in body weight between reversal and male chicken. *p* < 0.001. **b** Shank length of adult chicken. Asterisks indicate statistically significant differences in shank length. *p* < 0.001. N.S. indicate not statistically significant differences in shank length. *p* > 0.05. **c** Physical and gonadal appearance of adult chicken. Left: secondary sexual characteristics of adult chicken. Hackle feathers are marked by black arrow head and wattles are marked by asterisk. Right: left gonadal appearance and inner structure of adult chicken. Late-stage spermatid can be observed in male seminiferous tubules (black arrow). Bar = 50 μm. **d** Ultrastructural analysis of spermatic ultrathin sections from reversal and male chicken. Clear acrosome (A), perforatorium (P) and nucleus (N) can be observed in male sperm with slightly waved plasma membrane (black arrow). Bar = 2 μm

We monitored the growth performance of the chickens, including determining their body weight and shank length, over a 30-week period and examined their reproductive tissue development at the age of 30 weeks (Fig. 3a, b). We found that the weights of the male and female chickens diverged at 9 weeks. Interestingly, the weight increase in the sex-reversed chickens was almost identical to that in the females and was significantly different than that in their male counterparts. Furthermore, as expected, the length of the shank in the sex-reversed chickens was also significantly shorter than that in the males and followed a similar growth pattern to that in the female chickens. These results suggested that the growth performance of the sex-reversed chickens was consistent with that of the female chickens and differed from that of their male counterparts.

At the age of 30 weeks, the sex-reversed chickens were divided into three different groups (Degree 1, Degree 2, and Degree 3) based on their secondary sexual characteristics, where the appearance of masculinized characteristics was increasingly evident from Degree 1 to Degree 3 (Fig. 3c). In male group, chickens grew larger combs and wattles and well-developed leg spurs, possessing typical hackle feathers and sickle feathers. The sex-reversed chickens in the Degree 3 group were almost identical in appearance to the males, with large combs, wattles, and leg spurs. However, the Degree 2 group exhibited an ambiguous appearance, showing neither typical male nor female secondary sexual characteristics, especially regarding feather patterns, and they developed much smaller leg spurs than the males. The Degree 1 group exhibited a classic female-type feather pattern, and these chickens were overtly female in appearance. Analysis of the reproductive systems of the sex-reversed chickens revealed that the gonads of the Degree 3 group were approximately symmetrical but much smaller and less ovoid in appearance than those of the males, and the left gonad was still connected with a profoundly regressed oviduct. In the Degree 2 group, the gonads were asymmetrical, and there were some small follicles on the left side with a larger degenerated oviduct. As expected, the gonads of the Degree 1 group were almost identical to those of the female group, and these chickens could lay eggs from the oviduct.

To further examine the inner structure of the gonads in the different groups, histological sections of the gonads were prepared and stained with haematoxylin and eosin (H&E) (Fig. 3c). The reproductive tissue of the male chickens exhibited clear testicular structures with well-organized seminiferous tubules containing late-stage spermatids. The gonads of the female chickens showed a typical thick cortex with oocyte-containing follicles of different sizes. In sex-reversed chickens from the Degree 1 to the Degree 3 groups, the cortex of the gonads gradually regressed and contained fewer follicles, and the female-type vacuolated medulla formed progressively dense tubule structures. However, we failed to identify any late-stage spermatids in the tubule-like structures regardless of the degree of sex reversal. These results suggested that fadrozole exerted a slight impact on growth performance but significantly influenced the differentiation of the secondary sexual characteristics and reproductive tissues of the sex-reversed chickens.

### Fertility analysis of adult chickens

To determine whether the chickens in the Degree 3 group could produce fertile sperm, we attempted to milk semen from the Degree 3 group chickens. Interestingly, we found that six sex-reversed chickens ejected semen-like secretions induced by dorsa‐abdominal massage, and only five ejected semen regularly. However, no fertilized eggs were obtained after artificial insemination (Additional file 4: Table S6). Based on these results, we wanted to determine the reason for the infertility in our sex-reversed chickens. We speculated that the high deformity rate and abnormal structure of the sperm might have influenced the fertility of the sex-reversed birds. Therefore, we compared the structure of the sperm between males and sex-reversed chickens via ultrastructure analysis (Fig. 3d). Sperm from the male chickens presented with intact acrosomes, perforators, and nuclei and showed a slightly waved plasma membrane in the postacrosomal region. However, in the sex-reversed chickens, there were fewer sperm in the semen, and the sperm failed to form acrosome structures. Together, these results suggested that treatment with fadrozole led to the maldevelopment of the gonads and the production of severely deformed sperm in the sex-reversed chickens.

### Transcriptional profiling of adult chicken gonads

**Fig. 4:**
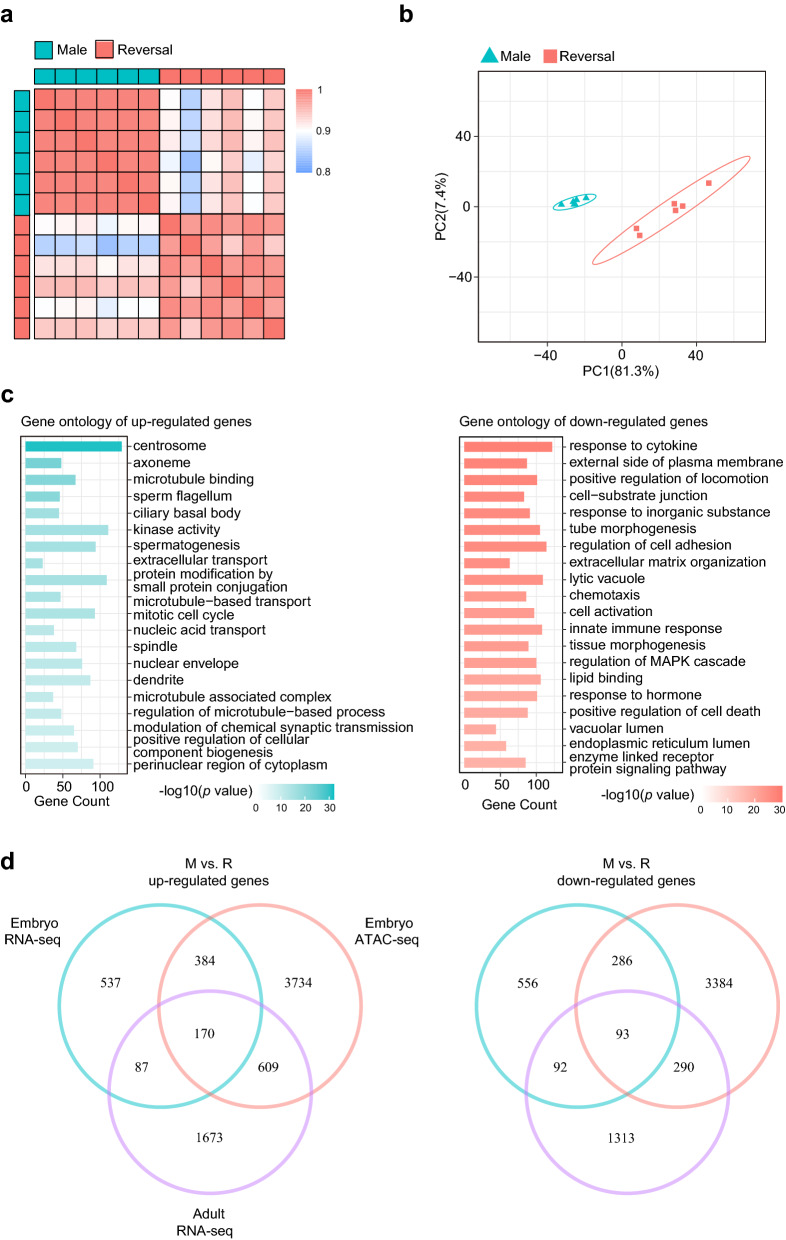
Analysis of RNA-seq data from adult chicken left gonads. **a** Correlation matrix of RNA-seq samples from adult chicken. **b** PCA plot of RNA-seq data from adult chicken left gonads. **c** Top 20 significantly enriched Gene Ontology terms for DEGs from adult chicken left gonads in M vs. R. **d** Venn diagrams show the shared and unique genes obtained from RNA-seq of embryo and adult chicken left gonads and ATAC-seq of embryo chicken left gonads in M vs. R

To explore the underlying mechanisms by which fadrozole affects adult chicken gonadal morphogenesis, and the regulatory basis of sperm maldevelopment in the sex-reversed chickens, RNA-seq was performed on samples taken from the left gonad of the 39-week-old chickens. We intentionally selected the six chickens from the sex-reversed group that could be milked to obtain a semen-like secretion, and we randomly chose six male chickens as controls. The mapping statistics were displayed in Additional file 4: Table S7. Six replicate samples in both groups showed significant correspondence (Fig. 4a). A PCA plot based on highly expressed genes showed a clear separation between the two groups (Fig. 4b).

By analysis and filtering, we detected a total of 4,327 DEGs, including 2,539 upregulated genes and 1,788 downregulated genes in the M vs. R comparison (Additional file 3: Fig. S3a). However, the majority of the DEGs were located on autosomes, and approximately 12% resided on the Z chromosome (Additional file 3: Fig. S3b). A functional enrichment analysis, as expected, indicated that the upregulated genes were primarily involved in sperm development processes, such as sperm flagellum, spermatogenesis, and the mitotic cell cycle pathways (Fig. 4c). The downregulated genes were enriched in cell activation, lipid binding and response to hormone pathways (Fig. 4c). These results suggested that in the adult sex-reversed chickens, the expression level of spermatogenesis-related genes was significantly lower than that in the male chickens.

To examine the influence of fadrozole on the development of the gonads and sperm during this 39-week period, we integrated RNA-seq data from both embryonic and adult gonads with the ATAC-seq data (Fig. 4d). As the Venn diagrams show, 257 DEGs were upregulated in both groups, and 170 of them harbored DARs that were enriched in 9 + 2 motile cilium, mitochondrial matrix, and regulation of the meiotic cell cycle pathways (Additional file 3: Fig. S3c). These DEGs contained *SPEF2*, *DNAI1*, and *TACR3,* which were related to flagellum development (Additional file 4: Table S8). However, 185 DEGs were downregulated, and 93 of them harbored DARs were involved in uterine development, urogenital system development and response to estradiol pathways (Additional file 3: Fig. S3c). These genes included *ESR1* and *FOXL2*, which were related to ovarian development (Additional file 4: Table S8). These results indicated that fadrozole failed to sufficiently upregulate genes related to testicular development and spermatogenesis and to fully downregulate genes required for ovarian and uterine development, leading to infertility in the sex-reversed chickens.

## Discussion

The sex determination and differentiation of chickens are the results of comprehensive complex regulatory mechanisms [19, 42]. In contrast to that in mammals, chicken gonadal sex differentiation is susceptible to the effect of hormones, especially oestrogen, which can be blocked by an aromatase inhibitor such as fadrozole, and this oestrogen reduction causes female-to-male sex reversal [28, 29, 34]. In addition, previous research has confirmed that hormones could exert a large impact on the binding of pioneer transcription factors (pTFs), which can regulate the process of chromatin remodeling and affect genomic accessibility, thus playing important roles in influencing the expression of downstream targeted genes [31, 50, 53, 54]. Although many studies have focused on the mechanism of chicken sex reversal, none have elaborated the gene-regulating network underlying sex reversal or the role of chromatin accessibility [35, 39]. As high-throughput sequencing and multiomics technology are further advanced, it will become more convenient and less costly to decipher the inner mechanism of chicken sex differentiation and reversal. In this research, we established a sex-reversed chicken model by injecting fadrozole into E3.5 chicken embryos and performed RNA-seq and ATAC-seq on E10 wild-type and sex-reversed chicken embryonic gonads to produce dynamic profiles of the transcriptome and chromatin accessibility landscapes during the female-to-male transition. Then, to explore the persistent impact of fadrozole on adult sex-reversed chickens, we fed the sex-reversed chickens to 39 weeks and recorded their growth during the whole feeding period. Through artificial insemination experiments, we determined that the sex-reversed chickens that we generated were infertile. We then performed ultrastructure analysis and RNA-seq to determine the cause of this infertility phenotype. Taken together, the results in the present work provide unique insights into the regulatory mechanisms underlying sex differentiation and gonadal development as well as infertility in sex-reversed chickens.

As expected, we found that more than 1000 DEGs (Clusters 1 and 5) were expressed following male patterns in the sex-reversed group after treatment with fadrozole. Among these DEGs, the female development-related genes *DDX4* and *TNFAIP6* were significantly downregulated in the sex-reversed chickens. *DDX4*, located on the Z chromosome, has been hypothesized to be a maternal determinant for the formation of the germline lineage [55]. *DDX4*-knockout female chickens were sterile and contained no detectable follicles post hatching, suggesting that *DDX4* is essential for oogonia and normal ovary development [56]. Therefore, the downregulation of *DDX4* in the sex-reversed chickens that we observed was likely related to the abnormal development of the ovary. *TNFAIP6* has been reported to regulate the process of ovulation and oocyte fertilization in mice, and our data suggested that this gene might also be involved in the sex determination and differentiation in chickens [56–59]. In contrast, several DEGs related to male gonadal development and sex hormone synthesis, such as *DMRT3* and *AKR1B10*, were upregulated in the sex-reversed group [60–62]. It is possible that changes in the expressional patterns of upstream regulatory factors elevated the expression of these DEGs in the sex-reversed chickens, while showing low expression in female chickens. According to the abovementioned findings, it is reasonable to conclude that fadrozole eventually led to the masculinized development of the bilateral gonads in the sex-reversed chickens and might have promoted the formation of sex-reversed phenotypes to some extent.

We also identified a group of DEGs (Clusters 2 and 6) that showed intermediate expression levels in the sex-reversed group. Fadrozole downregulated or upregulated the expression of these genes, almost to the expression levels in the male samples. However, in contrast to the DEGs in Clusters 1 and 5, the DEGs in Clusters 2 and 6 failed to reach similar levels of expression to those observed in males. Clusters 2 and 6 included *FOXL2* and *CYP19A1* as well as *DMRT1* and *AMH*, which likely play critical roles in the process of sex differentiation. In females, *FOXL2* is essential to ovarian development and can regulate aromatase (*CYP19A1*) expression by binding to its promoter, thereby contributing to the synthesis of oestrogen in the gonads [44, 63–65]. In this study, we used fadrozole to inhibit the activity of aromatase in the female chickens, and the expression of *CYP19A1* and *FOXL2* was significantly reduced. Previous studies have found that aromatase inhibitors could cause a *FOXL2* level reduction in females, suggesting that a feedback regulatory mechanism is established between the *CYP19A1* and *FOXL2* genes, which was consistent with our work [44]. Indeed, oestrogen and its receptor were highly expressed in the left female gonad, but not in the right gonad, and caused the hyperproliferation of cells in the left gonad, which had regressed in the right gonad [66]. In E10 embryos, the left gonad of the sex-reversed embryos was much smaller than that of the female embryos. Downregulated *FOXL2* and *CYP19A1* likely caused a decrease in oestrogen in the left gonad with hypoproliferation in the sex-reversed group. Moreover, *DMRT1* was located on the Z chromosome and acted as a pTF to control the process of male sex development. Overexpression of *DMRT1* in the female embryo or knockdown of *DMRT1* in the male embryo led to the development of a sex-reversed phenotype [33, 41, 75]. Thus, the intermediate expression of *DMRT1* that we observed in the sex-reversed chickens, which was higher than that in the females and lower than that in the males, likely contributed to the altered gonadal development in response to fadrozole. Recent studies have shown that oestrogen suppressed *DMRT1* expression during chicken ovarian development [67]. In our study, fadrozole likely caused a reduction in the oestrogen level, relieving the suppressive effects of oestrogen on *DMRT1* and resulting in the upregulation of *DMRT1* in the sex-reversed chickens. *DMRT1* can regulate the expression of downstream genes, such as *AMH,* which in turn regulate the elongation of reproductive ducts and the formation of gonads in the embryonic period [68, 69]. In addition, we found a promising male sex-related gene, *TOX3*, that has been previously reported to be a marker gene of the Sertoli cell lineage [70]. *AMH* and *TOX3* exhibited an expressional pattern similar to that of *DMRT1* in our study. Both of the aforementioned genes were upregulated by the overexpression of *DMRT1* in the sex-reversed chickens, suggesting that *DMRT1* activated male development pathways [40, 71]**.** In summary, our results showed that fadrozole downregulated *CYP19A1* and *FOXL2* and upregulated *DMRT1* in sex-reversed chickens but did not fully change the expression levels of these genes to those observed in males. The intermediate expression levels of master regulators of sex differentiation that we observed might explain the incomplete sex reversal of chicken embryos and the maldevelopment of gonads in response to fadrozole.

Through our results, we also discovered a class of DEGs (Clusters 3 and 7) whose expression levels were not affected by fadrozole. According to our functional analysis, these DEGs, including *WNT4*, *GATA6*, and *STAR*, which were highly expressed, and *SOX9*, *NR5A2*, and *DNAI1,* which were expressed at low levels*,* were mainly enriched in gamete generation, reproductive structure development, response to hormone, and glomerulus development pathways. In female chickens, *WNT4* regulated the development of the ovarian cortex via the *WNT4*/*R-spondin1*/*β-catenin* signaling pathway, which was associated with the activation of *FOXL2* and *CYP19A1* [72–74]. *GATA6* governs the differentiation and proliferation of granulosa cells and was involved in the coregulation of steroid metabolism with *STAR*, including during the production of oestrogen [75–77]. Moreover, it seems that fadrozole did not influence the expression of germ cell development-related genes, which might explain the infertility of the sex-reversed chickens. When we focused on the DEGs in Cluster 7, we found a testis development-related gene, *SOX9*, and a luteinizing hormone synthesis regulation gene, *NR5A2* [69, 70, 78]. Interestingly, we identified a set of spermatogenesis-related genes, *DNAI1*, *SPEF2*, and *TACR3*, that maintained low expression levels in the sex-reversed chickens [79–83]. Considering the function of *DNAI1* and *SPEF2*, which regulate the development and movement of flagella, we speculated that the low expression of these spermatogenesis-related genes caused the maldevelopment of germ cells in the sex-reversed chickens [79, 82].

In general, we revealed that the expression of sex development-related genes in female, sex-reversed, and male chickens presented 3 distinct patterns. Further studies are needed to determine how fadrozole drives the various transcriptional changes in these groups of genes. By integrating RNA-seq with ATAC-seq data from the male, female and sex-reversed groups, we found that changes in the chromatin accessibility of several genes corresponded to changes in their expressional patterns. Importantly, for *DMRT1* in Cluster 6, we observed marked differences in the enrichment of the ATAC-seq signal in its locus among the three groups, with the highest chromatin accessibility signal in the male group corresponding to the highest transcriptional levels. *TOX3* carried male-biased DARs compared with the sex-reversed group and reversal-biased DARs compared with the female group, which aligned with its expressional pattern [77]. In contrast, *FGFR3*, in Cluster 2, promoting the proliferation and survival of germ cells, showed opposite trends to *TOX3* in terms of the expression level and chromatin accessibility [91]. In addition, corresponding to expressional patterns, the chromatin accessibility of *GATA6*, *STAR*, and *SPIRE1* in Cluster 3, as well as *DNAI1*, *SPEF2*, and *TACR3* in Cluster 7, showed no significant changes between the female and sex-reversed groups, suggesting that fadrozole failed to affect accessible regions on these genes and thus did not affect their expressional patterns. These results indicated that treatment with fadrozole altered accessible chromatin regions to induce the genes in Clusters 2 and 6 to be expressed at intermediate levels in the sex-reversed group.

Focusing on our ATAC-seq results, we found that the motifs associated with certain core TFs, such as *DMRT1*, *NR5A2*, and *GATA* family members, were differentially enriched among the three chicken groups. The binding motifs of *DMRT1* and *NR5A2* were significantly enriched in male-biased and reversal-biased DARs when compared with those in the female group. Previous studies proved that the overexpression of *DMRT1* increased *TOX3* expression and modulated the expansion of the steroidogenic lineage in embryonic chicken gonads, and *TOX3* overexpression in female gonads resulted in a significant reduction in *CYP17A1*-positive steroidogenic cells, which was consistent with the expressional patterns of *DMRT1*, *TOX3*, and *CYP17A1* in the female and sex-reversed chickens in our study [71]. Hence, it is reasonable to suggest that *DMRT1* is the upstream male sex development-related gene in chickens, which functions in part to antagonize female sex developmental pathways [41, 84]. Moreover, *NR5A2* has been shown to regulate the expression of *CYP19A1* in chickens, which might contribute to this antagonistic relationship [78]. In contrast, the binding motifs of *GATA* family members, especially in *GATA6*, were significantly enriched in female-biased and reversal-biased DARs compared with those in the male group. The loss of *GATA6* and *GATA4* resulted in failed ovulation and infertility, and negatively affected the development of granulosa cells [75, 76, 85]. Considering these results, we speculated that a group of genes regulated by *GATA* family members maintained female expressional patterns in the sex-reversed chickens. Overall, fadrozole might affect the binding of certain types of TFs, which would further influence the remodeling process of genomic accessible regions, expression of sex development-related genes, and acquisition of sex-reversed phenotypes.

To explore whether the sex-reversed chickens produce fertile sperm, we reared a flock of sex-reversed chickens. We found that these adult sex-reversed chickens showed different degrees of sex-reversed phenotypes, which was consistent with previous reports [34, 35]. In our study, sex-reversed chickens maintained the same growth performance as females, but clearly developed typical male feathers and secondary sexual characteristics. However, only Degree 3 chickens, with the more profound sex-reversed phenotypes, produced semen. When female chickens were artificially inseminated with this semen, however, the eggs could not be fertilized, indicating that the sex-reversed chickens in our study were infertile. After dissection, all these different sex-reversed chickens showed ovotestes to different degrees. When compared to those of the females, the gonads in the Degree 3 chickens were almost symmetric, but we did not identify sex cords with clear outlines in them. In addition, the sperm in the Degree 3 chickens was greatly deformed, corresponding to that described in previous studies [38, 90]. These results indicated that the infertility of sex-reversed chickens was due to the abnormal structure of their reproductive organs, which did not successfully develop into functional testes and provide suitable environment for the differentiation of sperm. Moreover, RNA-seq of samples from adult male and Degree 3 sex-reversed chickens showed that DEGs related to sperm flagellum, spermatogenesis, and mitotic cell cycle pathways were downregulated in the sex-reversed chickens, which conformed with the abnormal phenotype of the sperm in the sex-reversed chickens. In particular, we identified three candidate DEGs, *DNAI1*, *SPEF2*, and *TACR3*, that maintained constant low expression levels from embryonic to adult stages of development and were not affected by treatment with fadrozole, which suggested that the expressional patterns of these genes in spermatogenesis-related cell lineages were constant and could not be easily changed. Hence, we considered that consistent low expression of several spermatogenesis-related genes might influence the differentiation of sperm in sex-reversed chickens. This means inherent female gonadal cell types and their gene expression profiles were difficult to be changed by fadrozole, which also contributed to the infertility of the sex-reversed chickens.

## Conclusions

In this study, we presented high-resolution transcriptome and chromatin accessibility landscapes of embryonic gonads in sex-reversed chickens. Our findings clearly illustrated that fadrozole influenced the expression of a set of key sex-related genes, including *FOXL2*, *CYP19A1*, *DMRT1*, and *SOX9,* and confirmed the central role of oestrogen in female gonadal differentiation. In addition, we found that fadrozole affected the expressional pattern of several promising genes, such as *FGFR3* and *TOX3*, by regulating their chromatin accessibility in sex-reversed chickens. Furthermore, we recorded the development of secondary sexual characteristics and the body growth of sex-reversed chickens, and analyzed the mechanisms underlying their infertility, which might have resulted from the low expression of several gametogenesis-related genes, such as *SPEF2*, *DNAI1*, and *TACR3*. Overall, our findings enable a better understanding of gonadal development in sex-reversed chickens and facilitate the construction of a gene regulatory network for chicken sex determination and differentiation. In addition, our study can serve as a potential guide for researching sex modulation in the poultry industry and treatment of human sex disorders.

## Methods

### Ethics statement

All animal experiments were approved by the Animal Welfare Committee of China Agricultural University (AW80802202-1–1) and performed in accordance with the protocol outlined in the “Guide for Care and Use of Laboratory Animals” (China Agricultural University).

### Embryo gonad collection and sex-reversed flock establishment

A total of 120 fertilized eggs from a population of commercial pure line White Leghorn and 1335 fertilized eggs from a pure line of sex-linked dwarf chickens, raised in the Experimental Base of Poultry Genetic Resources and Breeding, College of Animal Science and Technology, China Agricultural University, were disinfected and incubated at 37.8 °C and 65% relative humidity. On E3.5 (HH21), the eggs were randomly divided into either the control group or treated group. The air sac of the eggs in the treated group were injected with 100 μL PBS containing 1.0 mg Fadrozole (Selleck, Houston, America). After injection, the holes were sealed with paraffin, and the eggs were reincubated.

On E10 (HH36), the left gonads of the White Leghorn chicken embryos were dissected from the mesonephros and halved. The remaining bodies were collected to identify sex using *CHD1* primers with a direct PCR kit (TransGen Biotech, Beijing, China) (Additional file 4: Table S9) [86]. In addition, the gonads of all the genetic females from the treated group (reversal) and eight biological replicate samples of genetic males and females from the control group were prepared for the following steps.

Eggs from the sex-linked dwarf chickens were reincubated until birth. All chickens were reared in a brooder for 6 weeks and then transferred to wired cages. Water and food were provided ad libitum. At the age of 2 weeks, the whole blood of each chick, collected from the wing vein using a syringe, was used to identify the genotype using *CHD1* primers. Based on the genotype, the reversal group (*n* = 285), male group (*n* = 44), and female group (*n* = 26) were culled for further research. The body weight of each group at the ages of 4 weeks, 9 weeks, 13 weeks, 17 weeks, 20 weeks and 30 weeks was measured using an electronic scale by sampling or full measurement.

### Histological analysis and adult gonads collection

At the age of 30 weeks, chickens from the different groups were randomly selected for sacrifice, and the left gonads were dissected for histological analysis. Tissues were placed and fixed in testicular fixative (Servicebio, Wuhan, China), embedded in paraffin, sliced, and stained with hematoxylin and eosin for microscopic examination. At the age of 39 weeks, chickens from the male group and reversal group were sacrificed, and the left gonads were divided into two parts. Each group had six biological replicate samples. One part of each sample was placed in testicular fixative for histological analysis, and the other was used to extract total RNA.

### Sperm ultrastructure analysis

Semen was collected from the male group and reversal group by dorsa‐abdominal massage, placed in 2.5% glutaraldehyde fixative (prepared with 0.1 mol/l phosphoric acid buffer, pH = 7.2) and stored at 4 °C for 12 h. Subsequently, the semen was washed three times in 0.1 mol/l phosphoric acid buffer for 10 min and fixed with 1% osmic acid fixing solution (prepared with 0.1 mol/l phosphoric acid buffer, pH = 7.2) at 4 °C for 2 h. After washing with 0.1 mol/l phosphoric acid buffer three times, the samples were dehydrated by being passed through an ethanol series (50%, 75%, 95%, 100%) and through pure propylene oxide for 10 min. Then, the products were processed by three types of mixed solutions of propylene oxide and Spurr's resin in different ratios, followed by embedding into Spurr's resin and polymerizate at 70 °C for 14 h. Semi‐thin sections were stained with uranyl acetate and citromalic acid lead. Ultrathin Sections (70 nm) were placed on nickel grids, and a transmission electron microscope (HT7800, HITACHI, Japan) was used to analyze the sperm ultrastructure.

### RNA isolation, library preparation and sequencing

One part of the left gonad from embryonic chickens and the left gonad from 39-week-old adult chickens were placed in RNAwait (G-CLONE, Beijing, China) and stored at − 20 °C. The total RNA was extracted using an RNA extraction kit (TianGen Biotech, Beijing, China) following the manufacturer’s instructions. A NanoDrop 2000 spectrophotometer (Thermo Fisher Scientific, Waltham, United States) was used to evaluate the concentration and purity of the total RNA, and the integrity of the RNA was detected using an Agilent 2100 Bioanalyzer (Agilent Technologies, Santa Clara, United States). To construct the libraries, reverse transcription and amplification of the transcripts were performed using a Hieff NGS® Ultima Dual-mode mRNA Library Prep Kit (Yeasen Biotechnology, Shanghai, China). The libraries were sequenced on the Illumina NovaSeq 6000 platform.

### ATAC library preparation and sequencing

The other part of the left gonad from the chicken embryos was placed in a cryopreservation tube with preservation solution (DMEM/F12 supplemented with 10% fetal bovine serum and 10% DMSO). The samples were immediately placed in a cryopreservation box at 4 °C for 1 h and then stored at -80 °C overnight. We chose three replicate samples from each group to perform further steps. The viable cells from the gonads were pelleted at 500 RCF for 5 min at 4 °C in a fixed angle centrifuge, and the supernatant was aspirated. Fifty microliters of cold ATAC-resuspension buffer (containing 0.1% NP40, 0.1% Tween-20, and 0.01% digitonin) was added and mixed well. After incubating on ice, washing with ATAC–RSB, and centrifuging to collect the supernatant, the pellet nuclei were resuspended in 50 μL of the transposition mixture (containing 25 μL 2 × TD buffer, 2.5 μL transposase, 16.5 μL PBS, 0.5 μL 1% digitonin, 0.5 μL 10% Tween-20, 5 μL H2O) and incubated at 37 °C for 30 min. Then, the transposed fragments of DNA were used for PCR amplification. The PCR products were quantified with the KAPA Library Quantification Kit (Kapa Biosystems, Boston, United States) and then sequenced on the Illumina Novaseq 6000 platform.

### RNA-seq data analysis

Paired-end reads were aligned to the chicken reference genome (GRCg6a) by HISAT2 with default parameters followed by HTSeq-count to count the reads mapped to the genome [87, 88]. Then, a count matrix was used as the input to identify the DEGs between different groups by the DESeq2 package [89]. Candidate genes with greater than 1.5-fold changes at adjusted *p* values < 0.05 were considered to be significant DEGs.

### ATAC-seq data processing

The ATAC-seq reads were mapped to GRCg6a by BWA with default parameters [90]. Samtools was used to convert the SAM files to the BAM format, and PCR duplicates were cleared by the Picard MarkDuplicates option to generate filtered BAM files [91]. Then, the peaks were identified by MACS2 using the filtered BAM files with the parameters described in a previous article [86, 92]. All alignment files were scaled to Reads Per Kilobase per Million mapped reads (RPKM)-normalized read coverage files by deepTools [93]. The DESeq2 package was applied to the RPKM values to compare binding profiles between distinct groups in an unbiased manner to estimate the library size factors. The DARs were identified by DESeq2 with a fold change less than 1.5 and *q* value < 0.05. Motifs were detected by the HOMER tool (http://homer.salk.edu/homer/motif/), and the BEDtools suite (https://bedtools.readthedocs.io/en/latest/content/bedtools-suite.html) was used to test overlap and enrichment between different groups.

### Functional annotation

Using BioMart, we identified homologs of chicken DEGs in humans. Functional analysis of these homologs was performed by the Metascape online tool (http://metascape.org). The Gene Ontology (GO) terms containing biological process, cellular component, and molecular function categories were enriched with default parameters.

### Statistics and reproducibility

Statistical analyses were calculated by SPSS software (version 25.0; IBM, Chicago, IL, United States). The data were shown as the mean ± SD (standard deviation) analyzed using a two-tailed Student’s *t* test. At least three replicates were conducted in multiple independent experiments. The number of independent experiments was; seven for embryonic male gonad, twenty-three for embryonic reversal gonad, eight for embryonic female gonad in RNA-seq; three for embryonic gonad in each group in ATAC-seq; six, respectively, for adult male and reversal gonad in RNA-seq. In addition, 44 for adult male, 285 adult reversal, 26 for adult female were used in growth performance measurement. For multiple group comparisons, ordinary one-way ANOVA followed by Tukey’s multiple comparison test was performed. Statistical significance was shown as followed; ***p < 0.001, **p < 0.01, *p < 0.05.

## Supplementary Information

**Additional file 1: Fig. S1.** Analysis of DEGs from embryo chicken left gonads. **a** Correlation matrix of RNA-seq samples from embryo chicken. **b** Percent of DEGs and chromosomal allocation of embryo chicken left gonads in three pairwise comparisons. Since male chicken do not have W chromosomes, the expressed genes on W chromosomes are marked as “down-regulation” in M vs. F and M vs. R. **c** Volcano plots of DEGs number in three pairwise comparisons. **d** Venn diagrams show the shared and unique DEGs obtained from RNA-seq in three pairwise comparisons.

**Additional file 2: Fig. S2.** Analysis of DARs from embryo chicken left gonads**. a** Correlation matrix of ATAC-seq samples from embryo chicken. **b** Chromosomal allocation of DARs from embryo chicken left gonads in three pairwise comparisons. Since male chicken do not have W chromosomes, the accessible regions on W chromosomes are marked as “down-regulation” in M vs. F and M vs. R. Left: the number of DARs. Right: the percent of DARs. **c** Distribution of DARs in the genome from embryo chicken left gonads in three pairwise comparisons. Left: the number of DARs. Right: the percent of DARs.

**Additional file 3: Fig. S3.** Analysis of DEGs from adult chicken left gonads. **a** Volcano plot of DEGs number in M vs. R. **b** Chromosomal allocation of DEGs from adult chicken left gonads in M vs. R. Since male chicken do not have W chromosomes, the expressed genes on W chromosomes are marked as “down-regulation” in M vs. F and M vs. R. Left: the number of DEGs. Right: the percent of DEGs. **c** Top nineteen significantly enriched Gene Ontology terms for genes from embryo and adult chicken left gonads in M vs. R.

**Additional file 4: Table S1.** Summary of sequencing quality and reads alignment statistics of embryo gonads RNA-seq data. **Table S2.** DEGs in each cluster. **Table S3**. Summary of sequencing quality and reads alignment statistics of embryo gonads ATAC-seq data. **Table S4.** Summary of overlapping genes of embryo gonads RNA-seq and ATAC-seq data. **Table S5.** Record of hatching and genotype identification. **Table S6.** Record of artificial fertilization experiment. **Table S7.** Summary of sequencing quality and reads alignment statistics of adult gonads RNA-seq data. **Table S8.** Summary of common genes between embryo and adult gonads RNA-seq data and embryo gonads ATAC-seq data. **Table S9.** Primers used for sex PCR.

## Data Availability

The original contributions presented in the study are publicly available. These data can be found here: National Center for Biotechnology Information (NCBI) BioProject database under accession number PRJNA867032.

## Abbreviations

TFs: Transcription factors
E: Embryonic Day
HH: Hamburger Hamilton Stage
PGCs: Primordial germ cells
RNA-seq: RNA sequencing
ATAC-seq: Assay for Transposase Accessible Chromatin sequencing
DEGs: Differentially expressed genes
DARs: Differentially accessible regions
H&E: Hematoxylin and eosin
CASI: Cell autonomous sex identity
A: Acrosome
P: Perforatorium
N: Nucleus
