# Supplementary figures and images for: ATAC-seq and RNA-seq analysis unravel the mechanism of sex differentiation and infertility in sex reversal chicken
Source: Epigenetics Chromatin. 2023 Jan 9;16:2. doi: 10.1186/s13072-022-00476-1 (PMC9827654; doi:10.1186/s13072-022-00476-1)

**a**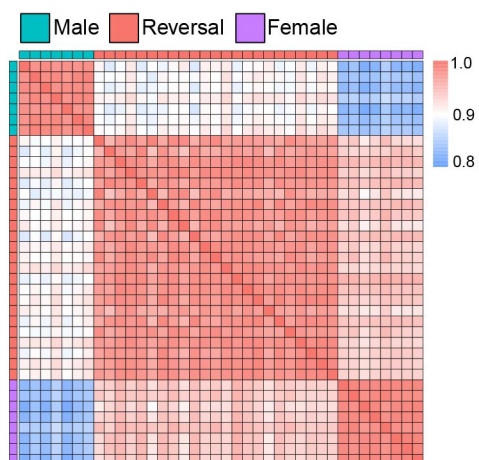**b**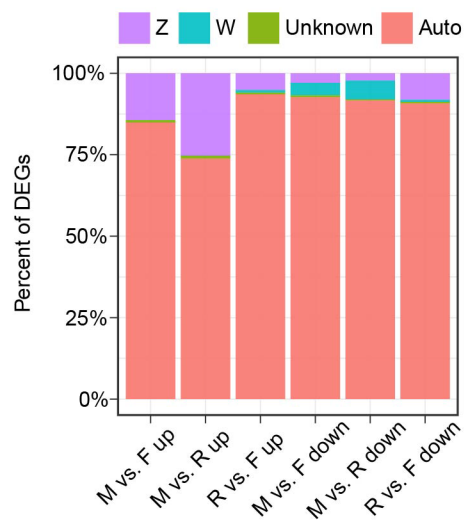**c**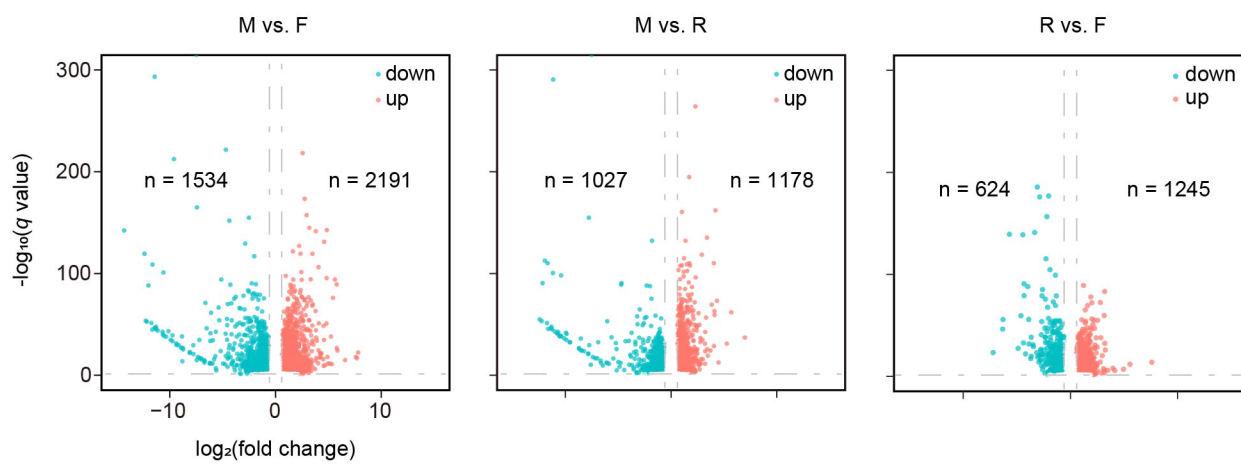**d**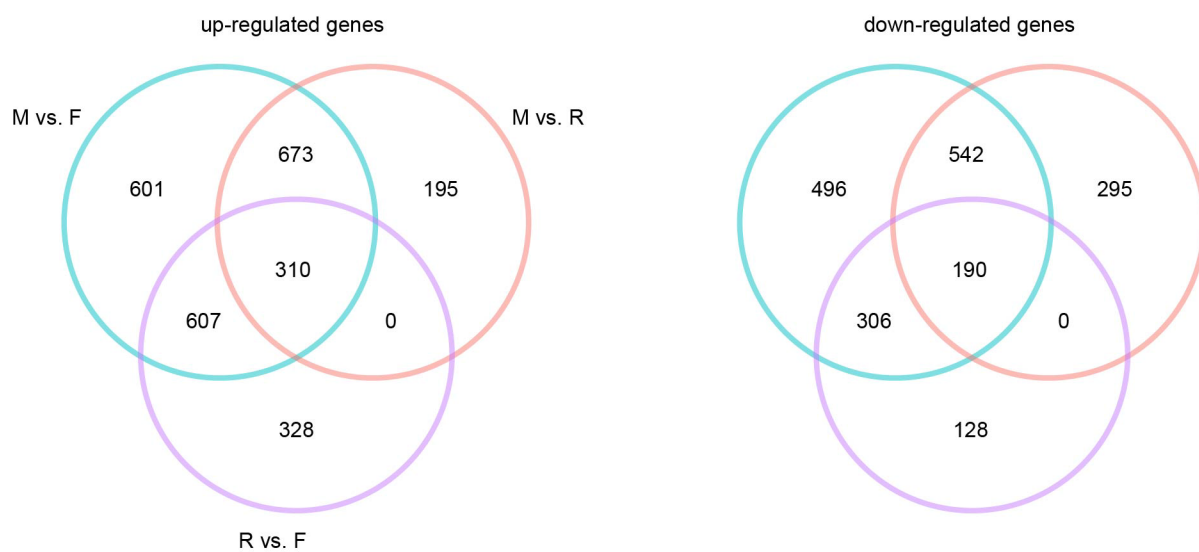

Supplement: Supplementary file 1 — Additional file 1: Fig. S1. Analysis of DEGs from embryo chicken left gonads. a Correlation matrix of RNA-seq samples from embryo chicken. b Percent of DEGs and chromosomal allocation of embryo chicken left gonads in three pairwise comparisons. Since male chicken do not have W chromosomes, the expressed genes on W chromosomes are marked as “down-regulation” in M vs. F and M vs. R. c Volcano plots of DEGs number in three pairwise comparisons. d Venn diagrams show the shared and unique DEGs obtained from RNA-seq in three pairwise comparisons. [file 13072_2022_476_MOESM1_ESM.pdf]

**a**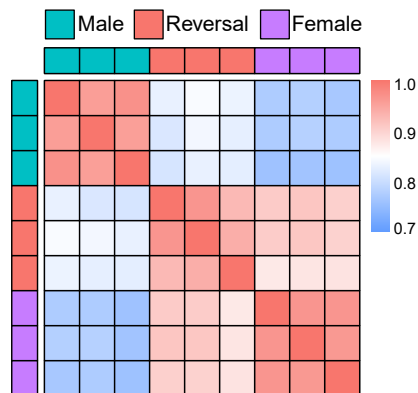**b**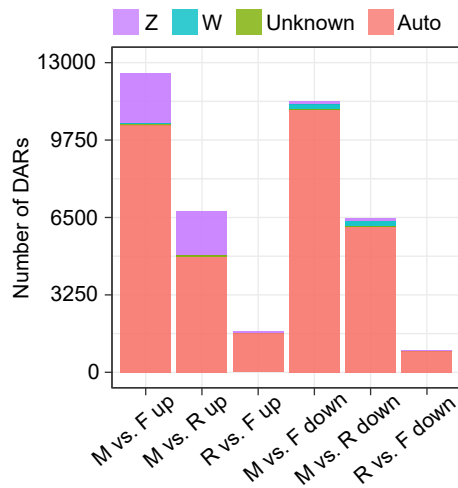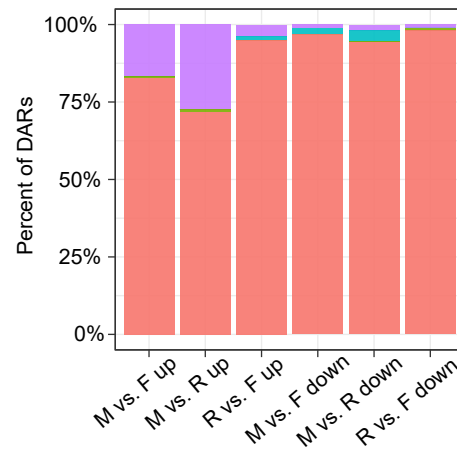**c**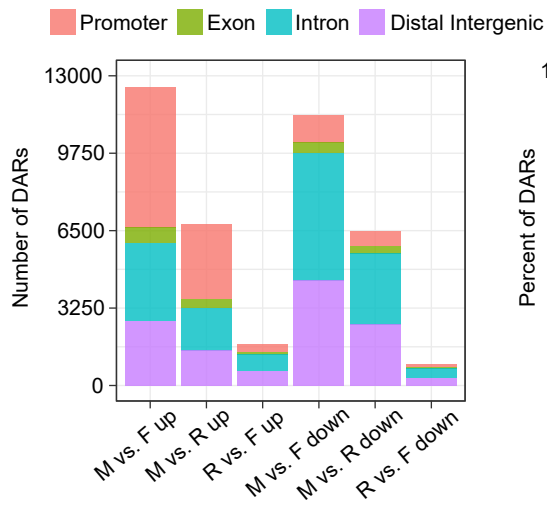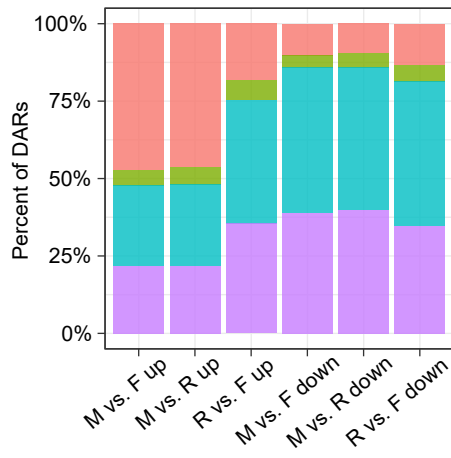

Supplement: Supplementary file 2 — Additional file 2: Fig. S2. Analysis of DARs from embryo chicken left gonads. a Correlation matrix of ATAC-seq samples from embryo chicken. b Chromosomal allocation of DARs from embryo chicken left gonads in three pairwise comparisons. Since male chicken do not have W chromosomes, the accessible regions on W chromosomes are marked as “down-regulation” in M vs. F and M vs. R. Left: the number of DARs. Right: the percent of DARs. c Distribution of DARs in the genome from embryo chicken left gonads in three pairwise comparisons. Left: the number of DARs. Right: the percent of DARs. [file 13072_2022_476_MOESM2_ESM.pdf]

**a**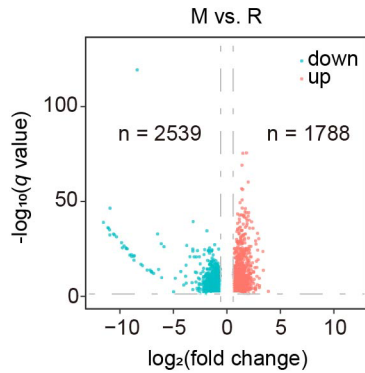**b**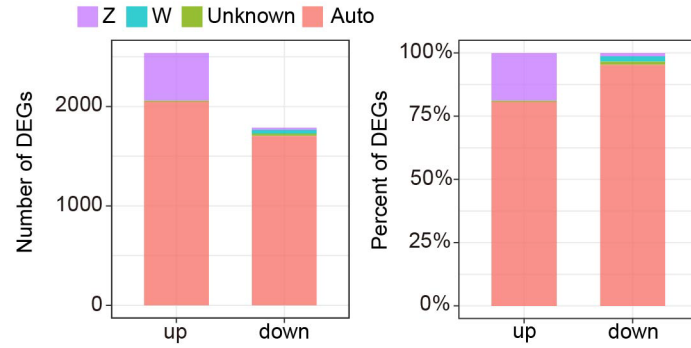**c**

Gene ontology of up-regulated genes

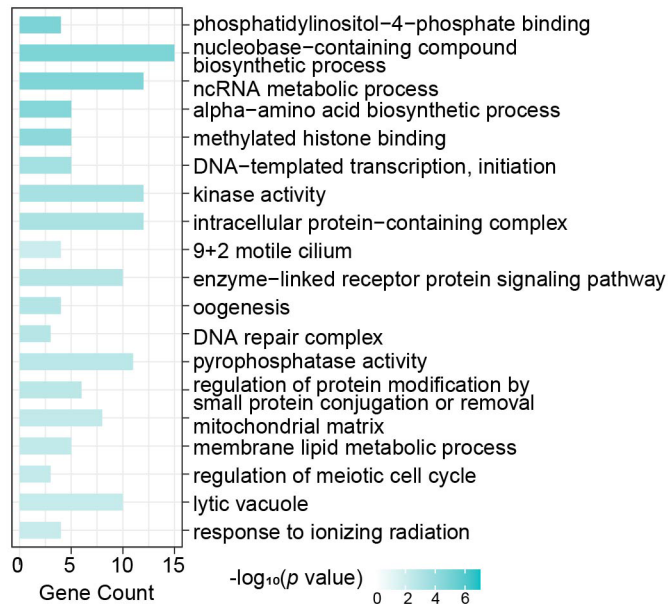

Gene ontology of down-regulated genes

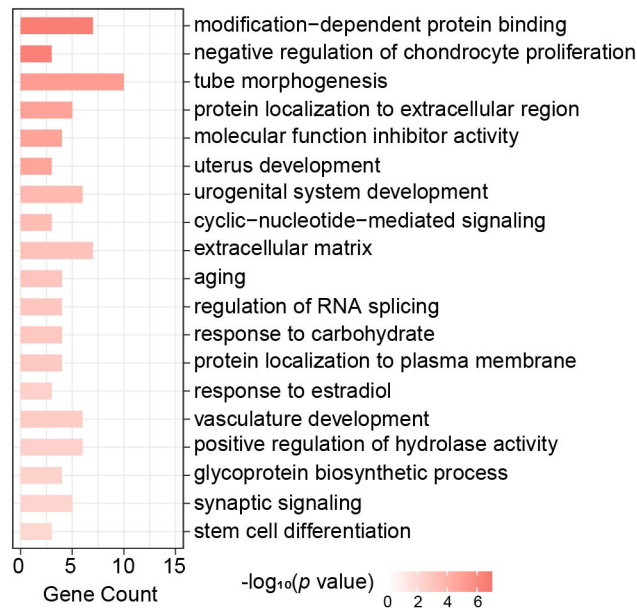

Supplement: Supplementary file 3 — Additional file 3: Fig. S3. Analysis of DEGs from adult chicken left gonads. a Volcano plot of DEGs number in M vs. R. b Chromosomal allocation of DEGs from adult chicken left gonads in M vs. R. Since male chicken do not have W chromosomes, the expressed genes on W chromosomes are marked as “down-regulation” in M vs. F and M vs. R. Left: the number of DEGs. Right: the percent of DEGs. c Top nineteen significantly enriched Gene Ontology terms for genes from embryo and adult chicken left gonads in M vs. R. [file 13072_2022_476_MOESM3_ESM.pdf]
